# Case report: a rare *BRCA1 de novo* variant in a female with breast cancer

**DOI:** 10.1186/s13053-026-00333-2

**Published:** 2026-03-12

**Authors:** Carlotta Dencker, Vincent Strehlow, Bahriye Aktas, Johannes Lemke, Julia Hentschel

**Affiliations:** 1 Institute of Human Genetics, University of Leipzig Medical Center, Philipp-Rosenthal-Str. 55, 04103 Leipzig, Germany; 2 Department of Gynaecology, University of Leipzig Medical Center, Leipzig, Germany

**Keywords:** *De novo*, Breast cancer, *BRCA1*, HBOC

## Abstract

**Background:**

Breast cancer is the most common cancer among women worldwide. While lifestyle factors contribute to rising incidence, 5–14% of cases result from pathogenic variants in core susceptibility genes such as *BRCA1*, which also increases ovarian cancer risk and, in men, prostate cancer risk. *BRCA1* variants are typically autosomal dominant, making family history a key criterion for genetic testing under guidelines like HBOC or NCCN. Although usually inherited, *de novo BRCA1* pathogenic variants occur rarely; only twelve cases have been reported. We present a young woman with breast cancer without a significant family history, and a pathogenic *de novo BRCA1* variant.

**Case presentation:**

We report a 37-year-old woman with HER2-positive, ER/PR-positive invasive breast cancer without relevant family history. After imaging-confirmed T1cN0M0 disease, she received neoadjuvant Her2-targeted chemotherapy, breast-conserving surgery, postneoadjuvant trastuzumab emtansine, radiotherapy, and ongoing endocrine therapy. Genetic testing by Next Generation Sequencing revealed a *BRCA1* frameshift variant (NM_007294.4:c.1335_1336del, p.(Arg446Serfs*9)) which was classified as pathogenic per ENIGMA/ACMG guidelines. Absent from population databases and previously reported in cancer cases, it disrupts protein function. Cascade testing showed neither parent carried the variant; microsatellite analysis confirmed parentage, indicating a *de novo* pathogenic variant.

**Conclusion:**

A rare *de novo* *BRCA1* variant was identified in a young breast cancer patient. Such variants are likely underdiagnosed due to historical testing limitations and reliance on family history. This case highlights the importance of genetic testing and inclusion in hereditary cancer prevention programs, even without a family history.

**Supplementary Information:**

The online version contains supplementary material available at 10.1186/s13053-026-00333-2.

## Background

Breast cancer is the most common malignancy among women worldwide, with an incidence of 47 per 100.000 women [1]. 5-14.1% of breast cancer cases are attributable to pathogenic genetic variants in common breast cancer genes, with pathogenic variants in the *BRCA1* gene being a well-known cause [2, 3]. *BRCA1* variants are inherited in an autosomal dominant manner. Consequently, these variants are frequently associated with a notable family history of the disease, and family history remains a central criterion for genetic testing.

According to established guidelines (such as the HBOC (Hereditary Breast and Ovarian Cancer) or NCCN (National Comprehensive Cancer Network) criteria [4, 5]), individuals undergo genetic testing if they meet specific clinical or familial risk factors, including early-onset breast cancer, triple-negative phenotype, bilateral disease, or a family history of breast or ovarian cancer. A genetically confirmed pathogenic variant in one of the breast cancer susceptibility genes (e.g. *BRCA1*) qualifies patients to participate in intensive early detection program and consideration of risk-reducing surgery. Although most *BRCA1* variants are inherited, *de novo* variants can occur.

In the general population, the rate of *de novo* variants is estimated in a total of 98–206 *de novo* pathogenic variants per transmission [6]. The highest incidence of pathogenic *de novo* variants is observed in neurodevelopmental and developmental disorders, where up to 42% of individuals with severe, undiagnosed developmental disorders carry pathogenic *de novo* variants in coding sequences [7]. The incidence of pathogenic *de novo* variants in cancer is generally low, and specifically, the incidence of pathogenic *de novo* variants in *BRCA1* and *BRCA2* genes is 0.3% among *BRCA1*/*2* pathogenic variant carriers [8].

To date, only twelve cases of *de novo BRCA1* variants have been reported in the literature, often with unremarkable family histories [8–17]. In this case report, we describe another instance of a young woman with breast cancer who had no remarkable family history and was found to carry a pathogenic *de novo BRCA1* variant.

## Case presentation

### Patients presentation

We present a case study of a 37-year-old female patient who was diagnosed with an invasive carcinoma of the right breast in 2022. The family history was unremarkable with regard to genetic tumour predisposition syndromes, in particular breast and ovarian cancer (see Supplement Fig. S1). An initial ultrasound examination identified a tumour measuring 1.1 × 0.6 × 5.0 cm, a finding that was subsequently confirmed by magnetic resonance imaging. No additional lesions suggestive of metastatic disease were detected on imaging. The patient underwent neoadjuvant systemic therapy consisting of 12 cycles of paclitaxel combined with dual receptor blockade, followed by four cycles of epirubicin and cyclophosphamide chemotherapy. Surgical management entailed breast-conserving surgery (lumpectomy) with a sentinel lymph node biopsy. Histopathological evaluation revealed a tumour with 100% positivity for oestrogen receptor (ER) and progesterone receptor (PR). Human epidermal growth factor receptor 2 (HER2/neu) status was scored as 3 + by immunohistochemistry. The Ki-67 proliferation index was quantified at 35%. Tumour staging at diagnosis was clinical T1c N0 M0, with a histological grade of G3. Following surgery, the patient underwent adjuvant therapy with trastuzumab emtansine for approximately one year due to residual tumour disease, alongside ovarian suppression using gonadotropin-releasing hormone agonists and aromatase inhibitors. Additionally, adjuvant radiotherapy was administered to the right breast. Since November 2022, the patient has undergone adjuvant endocrine therapy, comprising letrozole and Goserelin in conjunction with neratinib for one year, a HER2-directed tyrosine kinase inhibitor.

### Genetic testing

Genetic counselling and germline genetic testing using Next Generation Sequencing (NGS) of the patient were performed in 06/2024 in another clinic, identifying a pathogenic heterozygous variant NM_007294.4:c.1335_1336del, p.(Arg446Serfs*9) in the *BRCA1*-Gene. We performed a diagnostic NGS panel analysis covering all known cancer-associated genes (including the most relevant HBOC Core-genes), using genomic DNA extracted from peripheral blood leukocytes to confirm the previously known variant. Panel analysis was performed using Next-Generation-Sequencing via TWIST targeted enrichment following by short read sequencing on a NovaSeq6000 (Illumina) without detecting a mosaic. Variant nomenclature followed HGVS guidelines, with genomic positions referenced to hg38 [18]. Variant classification adhered to ClinGen ENIGMA BRCA1/BRCA2 Expert Panel Specifications to the ACMG/AMP Variant Interpretation Guidelines for *BRCA1* v1.2 [19].

The deletion of the two nucleotides leads to a *frameshift* and consequently to a premature stop codon (ACMG criteria applied: PVS1, PM5_STR_PTC), most likely resulting in a missing or disrupted protein product. This variant does not occur in the general population (gnomAD: 0%, v.4.1.0, ACMG criterion applied: PM2_SUP) [20]. It is listed twice in the HGMD database (ID: CD044265) [21]. The variant was assessed as pathogenic 6 times in ClinVar (ID: 54205) [22].

The polygenic risk score (PRS) for breast cancer was 1.6071 (z-score), placing the patient at the 95th percentile of the reference distribution; however, this value is still considered within the normal range. The genetic ancestry was calculated as European. The breast cancer PRS was calculated based on 313 breast cancer–associated variants (BCAC313) as defined by Mavaddat et al. [23]. We calculated the PRS for the following reasons: For carriers of pathogenic variants in core breast cancer genes, the PRS can help further refine individual risk estimates [24–26]. The PRS can also provide information about the risk of developing cancer in the opposite breast [27]. At our centre, the PRS assessment is fully integrated into routine diagnostic care, allowing us to tailor risk management and counselling to each patient’s unique genetic profile.

Subsequently, the patient’s biological parents attended our genetics clinic for cascade testing to determine their carrier status for the familial *BRCA1* pathogenic variant in 2025. We performed the abovementioned NGS analysis to segregate for the *BRCA1*-variant. However, genetic testing of the patient’s biological parents did not reveal the familial *BRCA1* variant, indicating that the patient had a d*e novo* variant. Low level germ cell mosaicism cannot be excluded. To ensure the genetic parentage, we performed a microsatellite analysis. Six out of nine markers were informative for the confirmation of parenthood.

## Discussion

Since 1999, 12 *de novo BRCA1* pathogenic variants have been reported (Table 1). A striking aspect of *de novo* variants is the absence of a significant family history. While some patients reported limited cancer history in relatives (e.g., paternal or maternal cousins with breast cancer [8, 11, 12, 14, 16, 17] it is important to note that the presence of breast cancer in more distant relatives may reflect the overall high prevalence of breast cancer in the general population (13.2% in Germany), rather than indicating a hereditary cancer syndrome [28].

Although the variant was not detected in parental blood, the possibility of low-level parental mosaicism, particularly gonadal mosaicism, cannot be entirely excluded by testing blood samples. As the patient has no siblings, there are no additional individuals available for testing who could provide indirect evidence for or against parental mosaicism.

The presented patient underwent genetic testing despite not meeting the German HBOC criteria that would typically justify genetic diagnostic [29]. She was not diagnosed before the age of 36, did not present with triple-negative breast cancer, and had no notable family history of the disease. The reason for external testing two years after the initial diagnosis remains unclear.

In Germany, in cases of *de novo* variants in breast cancer genes, patients are generally only offered genetic testing once they already developed breast or ovarian cancer, since their family history is often unremarkable and preventive testing in relatives is not typically justified. Without testing, a *de novo* pathogenic variant would remain undetected, potentially delaying appropriate surveillance and risk reducing surgery (mastectomy and/or salpingo-oophorectomy) for both the patient and her children. Furthermore, the patient’s children are now eligible for genetic testing—an entitlement they would not have had if the pathogenic *BRCA1* variant had not been identified in the patient.

Broader access to comprehensive sequencing and inclusion in the German Consortium-HBOC surveillance program are essential to ensure that both patients and their families receive appropriate risk assessment, surveillance, and preventive care. With more comprehensive sequencing now available, the true frequency of *de novo BRCA1* pathogenic variants may be higher than the currently assumed 0.4% [8, 18]. To not oversee patients without positive family history, broadening of inclusion criteria for genetic testing can be discussed.

We present another rare case of a *de novo BRCA1* variant in a young breast cancer patient. Pathogenic *de novo BRCA1* variants, though rare, are clinically significant and may be underdiagnosed due to historical testing limitations and reliance on family history, as well as the lack of parental testing, especially in older patients where parents are often no longer available for analysis. Our case reinforces the need for genetic testing in all breast cancer patients with early onset or aggressive subtypes, regardless of family history.

**Table 1:** *De novo* variants in *BRCA1*, reported in the literature since 1999 and features of the affected individuals

Year, Reference	De novo Variant (NM_007294.4)*	Clinical Features (Age of diagnosis, Tumour identity)	Family History, age of diagnosis
1999 [9]	c.3770_3771del, p.(Glu1257fs*9)	< 40, invasive ductal BC high grade, pathogenic variant in *BRCA2-*gene (6174delT)	Father prostate cancer, age early 50s, pathogenic variant in *BRCA2-*gene (6174delT)
2009 [10]	c.5332 + 1G > A, p?	bilateral BC; 38, invasive ductal BC, grade 2, ER+; 43, invasive ductal BC grade 3, ER+, PR+	Maternal aunt BC, age 54
2011 [11]	Deletion Ex 1–12*	30, invasive ductal BC, grade 2, TNBC	None
2011 [12]	Complete *BRCA1* gene deletion*	Bilateral BC; 28, invasive ductal carcinoma; 37, medullary carcinoma	Paternal cousin BC 42, uncle prostate cancer
2012 [13]	c.3494_3495del, p.(Phe1165Cysfs*2)	52, BC; 53, ovarian cancer	None
2013 [14]	Deletion of *BRCA1* exon 16 (mosaic)*	Bilateral BC; 39, grade 3, TNBC; 47, ductal carcinoma in situ, grade 3, TNBC	Paternal cousin BC, age 30
2016 [8]	c.5468–2 A > G, p.?	39, ovarian cancer	2 paternal aunts BC, age 57, 75
2016 [8]	c.2296_2297del, p.(Ser766*)	31, BC	None
2016 [8]	Mosaic exons 1–13 deletion*	41, invasive ductal BC	2 sisters BC, age 62, 63; paternal grandmother BC, age 75
2017 [15]	c.5095 C > T, p.(Arg1699Trp)	32, invasive ductal BC, ER+, PR+, HER2-	None
2023 [16]	c.4065_4068del, p.(Asn1355Lysfs*10)	30, invasive BC, HR+, HER2-	Cousin of father BC, age 55
2023 [17]	c.121 C > T, p.(His41Tyr)	29, invasive ductal BC, high-grade, TNBC	Maternal grandfather prostate cancer, age 85

* All exon-spanning or whole-gene deletions are reported as described in the original publications. Due to the use of non-standardized or custom exon-numbering systems and the absence of unambiguous reference transcripts in several sources, a reliable concordance with exon numbering in NM_007294.4 cannot be ensured

## Supplementary Information

Below is the link to the electronic supplementary material.

Supplementary Material 1

## Data Availability

The data that support the findings of this study are available from the corresponding author upon reasonable request.

## Declarations

### Generative AI and AI-assisted technologies in the writing process

During the preparation of this work the authors used ChatGPT in order to improve language and readability of the work. After using this tool, the authors reviewed and edited the content as needed and take full responsibility for the content of the publication.

## Abbreviations

BC: Breast cancer
*BRCA1*: Breast cancer 1 gene
ER: Estrogen receptor
HBOC: Hereditary breast and ovarian cancer
HER: Human epidermal growth factor
NCCN: National comprehensive cancer network
NGS: Next generation sequencing
PR: Progesterone receptor
PRS: Polygenic risk score
TNBC: Triple negative breast cancer
