# Supplementary material for: Case report: a rare BRCA1 de novo variant in a female with breast cancer
Source: Hered Cancer Clin Pract. 2026 Mar 12;24:6. doi: 10.1186/s13053-026-00333-2 (PMC13063632; doi:10.1186/s13053-026-00333-2)
Supplement: Supplementary file 1 — Supplementary Material 1 [file 13053_2026_333_MOESM1_ESM.docx]

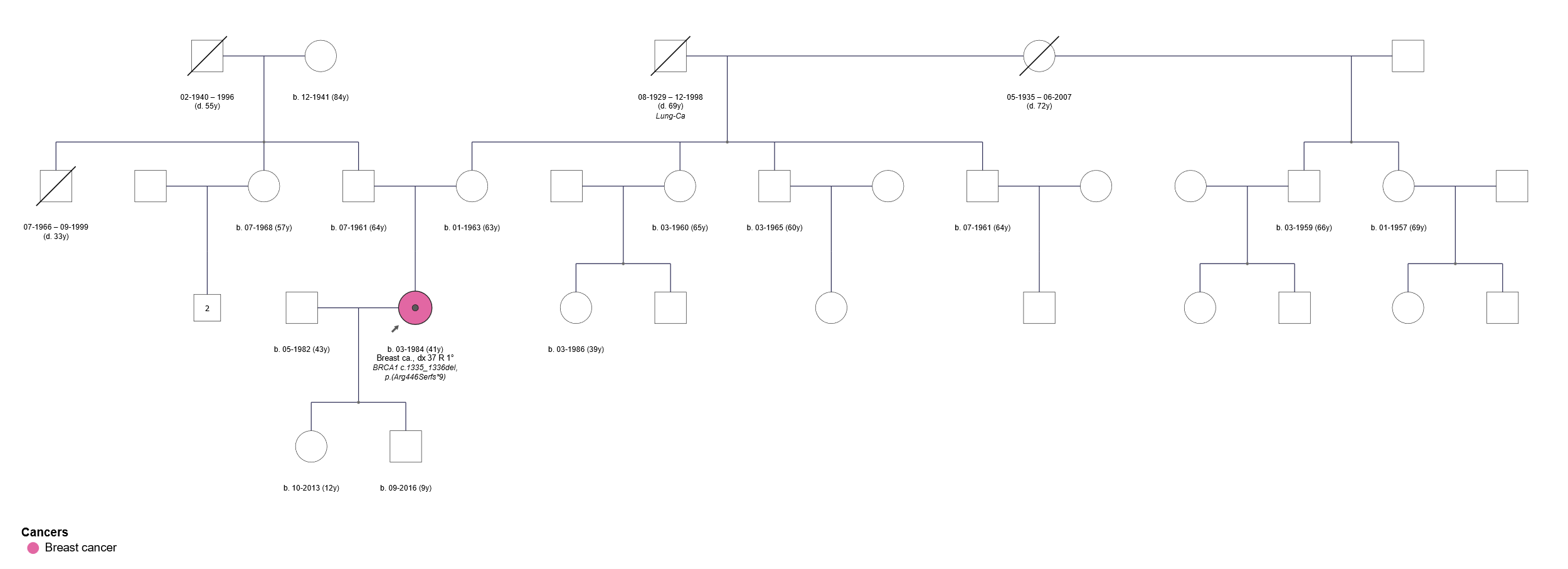
Supplementary Material

Figure S1: Pedigree of the affected individual (pink circle, indicated with an arrow); circles indicate females, squares indicate male family members, year of birth included wherever information was available.
